# Conjunction of potential G-quadruplex and adjacent cis-elements in the 5′ UTR of hepatocyte nuclear factor 4-alpha strongly inhibit protein expression

**DOI:** 10.1038/s41598-017-17629-y

**Published:** 2017-12-12

**Authors:** Shangdong Guo, Hong Lu

**Affiliations:** Department of Pharmacology, SUNY Upstate Medical University, Syracuse, NY 13210 United States

## Abstract

Hepatocyte nuclear factor 4-alpha (HNF4α) is a well established master regulator of liver development and function. We identified the *in vitro* presence of a stable secondary structure, G-quadruplex (G4) in the 5′ UTR of P1-HNF4A, the predominant HNF4α isoform(s) in adult liver. Our data suggest that the cooperation of G4 and the adjacent putative protein-binding sites within the 5′ UTR was necessary and sufficient to mediate a strong translational repression. This was supported by analysis of deleted/mutated 5′UTRs and two native regulatory single-nucleotide polymorphisms in the 5′UTR. Additional results indicated that G4 motifs in the 5′ UTRs of other liver-enriched transcription factors also inhibited protein expression. Moreover, pyridostatin, a G4 ligand, specifically potentiated the translational suppressing effect of P1-HNF4A-5′ UTR. In summary, the present study provides the first evidence of the presence of G4 in human P1-HNF4A-5′ UTR *in vitro*, and establishes a novel working model of strong inhibition of protein translation via interactions of G4 with potential RNA-binding proteins (RBPs). The protein expression of the tumor suppressor HNF4α may be inhibited by interactions of RBPs with the G4 motif in the 5′ UTR to promote cell proliferation during liver development and carcinogenesis.

## Introduction

Hepatocyte nuclear factor 4α (HNF4α) is a liver-enriched master regulator of liver development and differentiation^1^. HNF4α is essential for hepatocyte differentiation in fetuses^2,3^ and maintenance of liver function in adults^4–6^. Hepatic expression and/or activity of HNF4α are decreased markedly in severe cirrhotic livers, alcoholic liver disease, tumor necrosis factor-α-induced hepatotoxicity, and hepatoma progression^7–10^. HNF4α is down-regulated at protein levels during liver carcinogenesis in rats^11^. Hepatocellular carcinoma (HCC) is a primary malignancy accounting for 90% of liver cancers, the 3^rd^ leading cause of death from cancer worldwide^12^. Tumor development and cirrhosis cooperatively cause the destruction of the liver function. In this regard, an ideal treatment for HCC should not only suppress the progression of tumor cells but also improve the liver function. Interestingly, over-expression of HNF4α markedly inhibits liver carcinogenesis and liver fibrosis^13–15^. Thus, down-regulation of HNF4α is a major contributing factor to cirrhosis and liver cancer, whereas restoration of HNF4α can inhibit the development of the liver cancer and improve liver function simultaneously.

Like many important oncogenes and tumor-suppressors, HNF4α is an orphan nuclear receptor that lacks well-defined activating ligand; how to modulate these oncogenes and tumor-suppressors to treat cancer is a huge challenge. In view of the difficulty to directly modulate the protein activity, an alternative approach is to regulate their expression levels. The HNF4A gene uses two separate promoters P1 and P2, which encode overall 9 transcription isoforms (A1-A9) via alternative splicing. Products driven by the P1 promoter (HNF4A1-A6) are predominantly expressed in adult liver, whereas the P2 (HNF4A7-A9) products are prevalent in fetal liver and liver cancer^16^. Down-regulation of P1-HNF4A is a hallmark of HCC occurrence, during which P2-HNF4A are aberrantly elevated^17,18^. The 5′ UTR of mRNAs play a key role in regulating the translation of proteins. We found that the 5′UTR of several P1-HNF4A products, but not the P2-HNF4A, is highly G-C enriched and predicted to form complex secondary structures that are composed of the G-quadruplex (G4) and the stem-loop.

G4s consist of stacked planes of G-tetrads stabilized by hydrogen bonds via the Hoogsteen faces of the guanine residues. Compared to the double-stranded DNA, *in vitro*, many G4 DNA structures are thermodynamically more stable and their unfolding kinetics are much slower^19^. Recent studies demonstrate important roles of G4s in the regulation of DNA replication, gene expression, and telomere regulation^20,21^. For example, G4s are found *in vitro* within the promoter regions and have been implicated to play important roles in modulating the transcription of many oncogenes such as c-Myc, Bcl-2, and c-KIT^22–24^. However, studies of RNA G4s are limited. Compared to DNA G4s, RNA G4s are thermodynamically more stable and easier to be formed because RNAs are usually single-stranded^25–27^. RNA G4s are mainly localized within the 5′ and 3′ UTRs of messenger RNAs. G4 structures act as specific elements to regulate mRNA splicing, transcription termination, and translation^28,29^. In most cases, G4s suppress gene expression when localized within the 5′ UTR^30^. Such inhibitory effect can be explained by the blockage of the scanning stage during the translation initiation due to the stable complex secondary structures of G4s. The first example of translational repression by an RNA G4 located within the 5′UTR is the neuroblastoma RAS viral oncogene homolog (NRAS)^31^. Subsequent studies further demonstrate that the translation of oncogenes telomeric repeat binding factor 2 (TRF2) and matrix metallopeptidase 16 (MT3-MMP) are also suppressed by the G4 motifs within their 5′ UTRs^32,33^.

To date, the existence and importance of G4s in the 5′ UTRs of tumor suppressors have not been reported yet. The aim of this study was to characterize the putative G4 structures that we discovered in the P1-HNF4A-5′UTR. We used multiple approaches to identify the presence of G4s within the 5′ UTR *in vitro*. Moreover, via sequential deletion/mutation analysis of the luciferase reporters for the 5′ UTR, we found that the strong inhibitory effect requires the cooperation of G4 with the adjacent potential protein-binding sites to suppress the protein expression.

## Results

### P1-HNF4A-5′ UTRs markedly repress the reporter activity of luciferase and the protein expression of HNF4α1

**Figure 1:**
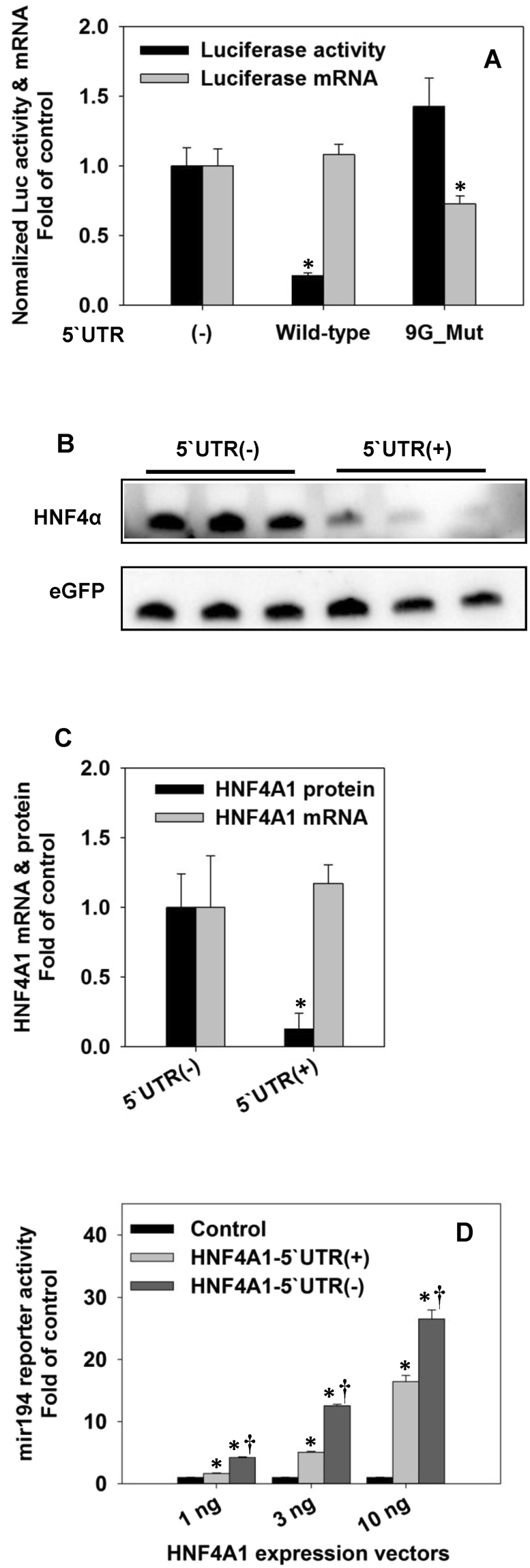
Effects of P1-HNF4A-5′UTR on luciferase activities and protein expression. **(A)** Quantification of the reporter activities and mRNAs of luciferase genes in HEK293 cells that were transiently transfected with the pRL-CMV control vector and luciferase reporter vectors for the wild-type and 9G-mutant P1-HNF4A-5′ UTRs. **(B)** Western blot of over-expressed HNF4α. HNF4A1 expression vectors with/without 5′UTR and an EGFP expression vector were co-transfected into HEK293 cells. The displayed bands for HNF4α and eGFP came from two sections of a single gel, revealed with different secondary antibodies. N = 3, mean ± SD. **(C)** Density analysis of Western blot and real-time PCR quantification of mRNA of HNF4A1 with/without 5′UTR. Both band density and mRNA expression of HNF4A1 were normalized to that of EGFP. **(D)** Dual-luciferase assay of effects of different HNF4α expression vectors on the activation of human miR-194 promoter in HEK293 cells. HEK293 cells in 96-well plate were co-transfected with miR-194 reporter vector, pRL-CMV vector, as well as 1, 3, and 10 ng HNF4α1 expression vectors. Y-axis represents normalized reporter activities. N = 4, mean ± SD. *p < 0.05 versus control group; ^†^p < 0.05 versus the corresponding 5′UTR(−) group.

The previous primer extension assay has identified an 89-nt long 5′UTR for P1-HNF4A^34^. According to the analysis of QGRS, a software that is extensively applied for the prediction of G4s^35,36^, this 5′UTR contains multiple successive “GGG” sets that allow itself to fold into different three-ring-G4s (three layers of the G-tetrads). Thus, the highly structured P1-HNF4A-5′ UTR has a great possibility to suppress the protein expression. To validate this hypothesis, we generated reporter vectors for the wild-type and a mutant (UTR_9G_Mut) P1-HNF4A-5′ UTR in which the potential formation of the three-ring-G4 was completely disrupted by replacing nine critical guanines with thymines. Results of dual-luciferase assay showed that the wild type 5′ UTR markedly reduced luciferase activity by 78%, whereas the G4-mutant 5′ UTR had no suppressing effect (Fig. 1A). Real-time PCR results showed that the wild type 5′UTR showed no decrease of luciferase mRNA (Fig. 1A). Taken together, our data clearly demonstrate that the 5′ UTR of P1-HNF4A dramatically represses the reporter activity, likely due to the G4-mediated inhibition of protein translation.

To determine the effect of the P1-HNF4A-5′ UTR on HNF4α protein expression, we transfected expression vectors for HNF4A1 cDNA with/without the 5′ UTR into HEK293 cells, which has no endogenous HNF4α. Results of Western blot showed that the 5′ UTR decreased HNF4α1 protein expression by 76% (Fig. 1B & C). In contrast, the 5′ UTR had no suppressing effect on the expression of HNF4A1 mRNA (Fig. 1C).

To elucidate the biological significance of the inhibition of P1-HNF4α protein expression by its 5′ UTR, we determined the effect of the 5′ UTR on HNF4α-mediated activation of the promoter of miR-194, a known HNF4α-target gene^37^ (Fig. 1D). Compared to the basal activity of miR-194, 1, 3, and 10 ng pcDNA3-HNF4A1-cDNA (without UTR) increased the reporter signal by 4.2, 13, and 27 fold, respectively. Meanwhile, equal amounts of pcDNA3-HNF4A1-5′ UTR (with UTR) only activate the miR-194 by 1.6, 5.2, and 16.8 fold, accordingly (Fig. 1D). Thus, P1-HNF4A-5′UTR plays a key role in limiting the protein expression and biological activities of HNF4α.

### Structure-activity relationship (SAR) studies of P1-HNF4A-5′UTR reveal the importance of the cooperation of the G4 with the potential protein-binding sites

**Figure 2:**
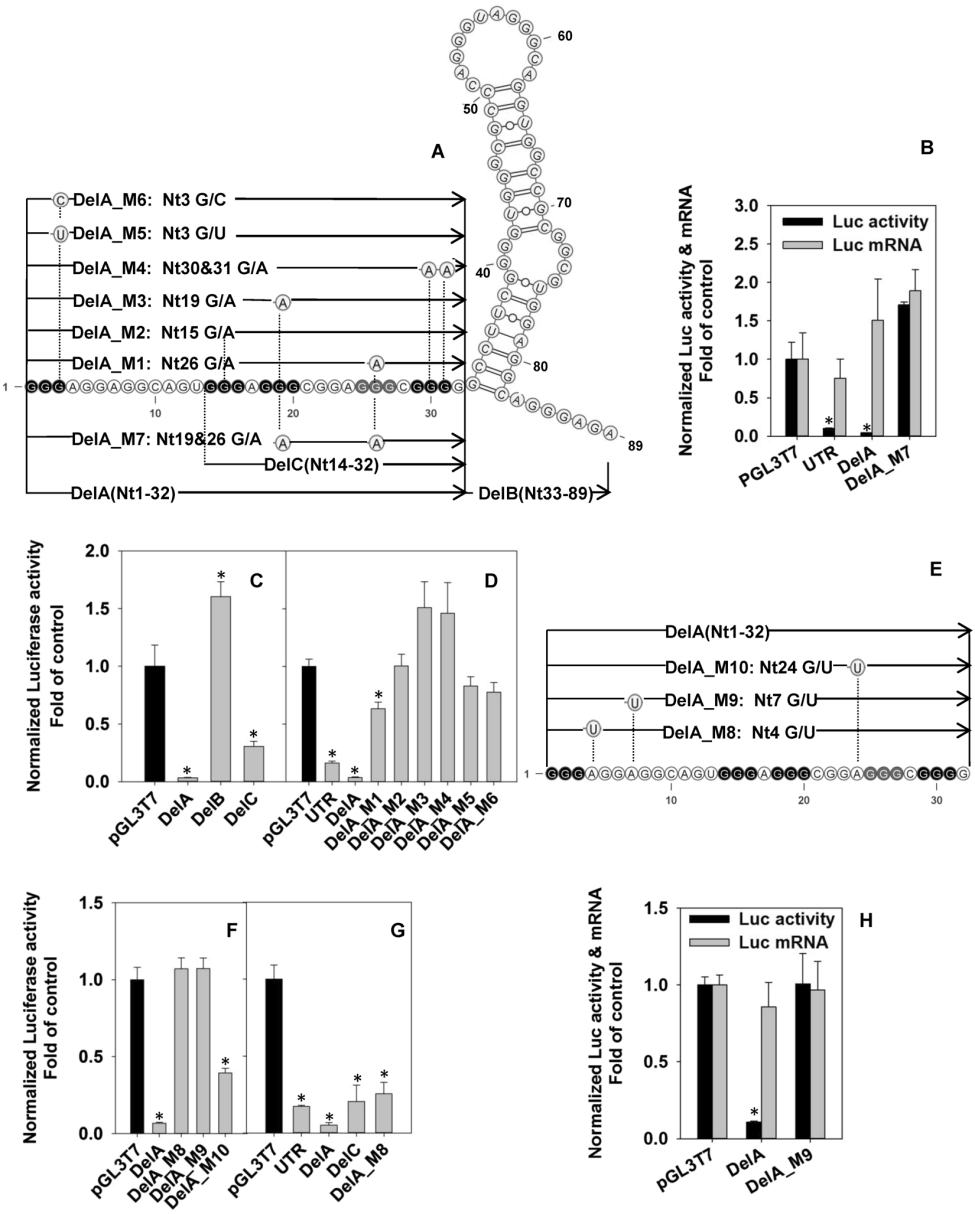
Structure-activity relationship (SAR) studies on the role of human P1-HNF4A-5′ UTR in the inhibition of protein expression of luciferase reporter gene in HepG2 cells. **(A**,**E)** Sequences and schematic structures of the full-length and deletion/mutation fragments of P1-HNF4A-5′UTR. **(B)** Luciferase reporter activities and mRNA levels of P1-HNF4A-5′UTR, DelA, and DelA_M7. **(C)** Dual-luciferase assay for DelA, DelB, and DelC. **(D**,**F)** Dual luciferase assay for the deletion/mutation reporter constructs for DelA. **(G)** Effects of deletion/mutation fragments of P1-HNF4A-5′ UTR on the luciferase reporter activities in the *in vitro* transcription/translation system. **(H)** Luciferase reporter activities and mRNA levels of DelA and DelA_M9 extracted from the cytosolic fraction of transfected cells. HepG2 cells were co-transfected with pGL3T7 firefly luciferase reporter vectors for deletion/mutation of P1-HNF4A-5′ UTR and the pRL-CMV control vector. N = 4, mean ± SD. *p < 0.05 versus pGL3T7 control. N = 3, mean ± SD. *p < 0.05 versus the pGL3T7 control.

The entire P1-HNF4A-5′UTR has the potential to form 3-ring-G4 structures in distinct conformations by using the various “GGG” sets within Nt1-32 (Fig. 2A). In addition, the formation of a stem-loop in Nt33-82 is predicted by RNAfold, a web-based software for analysis of RNA secondary structures^38^. To determine the roles of these structures, we constructed a set of reporter vectors by inserting the G4- and the stem-loop-containing motifs into the pGL3T7 backbone, a modified backbone based on pGL3-promoter (Promega) that contains a T7 promoter to drive the *in-vitro*-transcription/translation. Surprisingly, DelA, the G4 forming motif, solely caused an inhibitory effect (96% < control) that was slightly stronger than the full-length 5′UTR (91% < control) (Fig. 2B). In contrast, DelA_M7, a DelA mutant that loses the 3-ring-G4 motif, had no inhibitory effect (Fig. 2B). Real-time PCR analysis showed no difference in luciferase mRNAs, indicating that the substantial inhibitory effect of DelA was on protein translation (Fig. 2B). In contrast, DelB, the predicted stem-loop, had a stimulating effect (52% > control) on the reporter activity (Fig. 2C), which might be the cause of the slightly weaker inhibitory activity of the full-length 5′UTR than DelA. These data together indicate the predominant role of the upstream G4-forming motif (Nt1-32), namely DelA, in repressing protein translation.

Nt1-32 overall contains 5 sets of GGG, which allows the formation of the G4 in multiple possibilities. Thus, it is critical to determine the major conformation of G4 in Nt1-32 that causes the extremely strong inhibitory effect. We first tested DelC because it might be more stable than its peers due to the shortest side chains. Interestingly, DelC had much weaker inhibitory effect (70% < control) than DelA (Fig. 2C), indicating that the G4 formed in DelC alone is insufficient to induce a comparable inhibitory effect as DelA. We screened Nt1-32 for potential protein-binding sites with a web server RBPmap^39^. We found multiple motifs for recruiting RNA-binding proteins (RBPs), which include heterogeneous nuclear ribonucleoprotein H1/H2 (HNRNPH1/H2), HNRNPF, HNRNPA2, serine/arginine-rich splicing factor 1 (SRSF1), SRSF2, and SRSF9. Thus, we created different mutations on the G4-forming regions and the predicted RBP-binding sites to further investigate the SAR of the 5′UTR.

We first created reporter vectors by individually deleting/mutating 5 sets of GGG in DelA (DelA_M1-M6, Fig. 2A and Supplemental Table 1). In HepG2 cells (human hepatocellular carcinoma cell line), all constructs showed markedly diminished inhibitory effects: only DelA_M1 maintained a weak inhibitory effect (37% < control), whereas DelA_M2, DelA_M3, DelA_M4, DelA_M5 and DelA_M6 completely lost inhibitory effects (Fig. 2D). This suggests a less important role of the 4^th^ GGG (Nt 25-27), mutated in DelA_M1, than other GGGs in the G4 formation. Thus, the 1^st^ GGG (Nt1-3) might be used to form the G4 backbone.

We then assessed the effects of mutation of putative RBP-binding sites (DelA_M8-10, Fig. 2E) on translational suppression. Literature suggests that different RBPs may have distinct effects on G4 formation. Per RBPmap prediction, mutations DelA_M8/M9/M10 disrupt multiple predicted protein binding sites. Interestingly, DelA_M10 had partial loss of the inhibitory effect (61% < control) (Fig. 2F). Strikingly, with all the intact GGGs for G4 structures, DelA_M8 and DelA_M9 completely lost the inhibitory effect (Fig. 2F). Thus, the strong inhibitory effect of DelA requires both G4 and the RBP-binding sites.

Compared to living cells, the cell-free *in vitro* transcription/translation system from reticulocyte lysates has much lower concentration of K^+^ (communication with Promega), and thus the G4 formed in this *in vitro* system is chemically weaker than that in the intact cells; however, a comparable inhibitory effect by P1-HNF4A-5′UTR was observed (Fig. 2G). Additionally, by increasing the K^+^ concentration to 150 mM that is same with the living cells, the inhibitory effect of P1-HNF4A-5′UTR was strengthened remarkably (Fig. S3). These data suggest that the G4-unwinding capability of the *in-vitro*-translation system is much weaker than the living cells. Therefore, the *in-vitro*-translation system provides a special translational context that the G4 may become the predominant determining factor in the translational inhibition. As expected, all the tested G4-containing constructs had a strong inhibitory effect: UTR (83% < control), DelA (95% < control), DelC (81% < control), and DelA_M8 (76% < control) (Fig. 2G). The loss of inhibitory effect in HepG2 cells (Fig. 2F) but maintenance of strong inhibitory effect in the cell-free system (Fig. 2G) by DelA_M8, a binding-site mutant, suggests that the G4 within P1-HNF4A-5′UTR is sufficient to cause structural barrier for the basic translational machinery, which nevertheless may be overcome by the G4-unwinding factors in HepG2 cells. In this case, the RBPs bound to the side chains might be indispensable for stabilizing the entire G4 and maintaining the strong suppressing effect of the 5′ UTR. Some of the predicted RBPs, such as SRSF1 can shuttle the spliced mRNA between the nucleus and cytosol^40^. DelA caused a strong inhibition without affecting the cytosolic levels of mRNA (Fig. 2H), indicating that its inhibition of translation is not due to impaired nuclear export of mRNA.

### Protoporphyrin-IX (PPIX)-binding assay and circular dichroism (CD) spectrum further confirm the presence of the G4 in the 5′UTR RNA and deletion/mutation constructs

**Figure 3:**
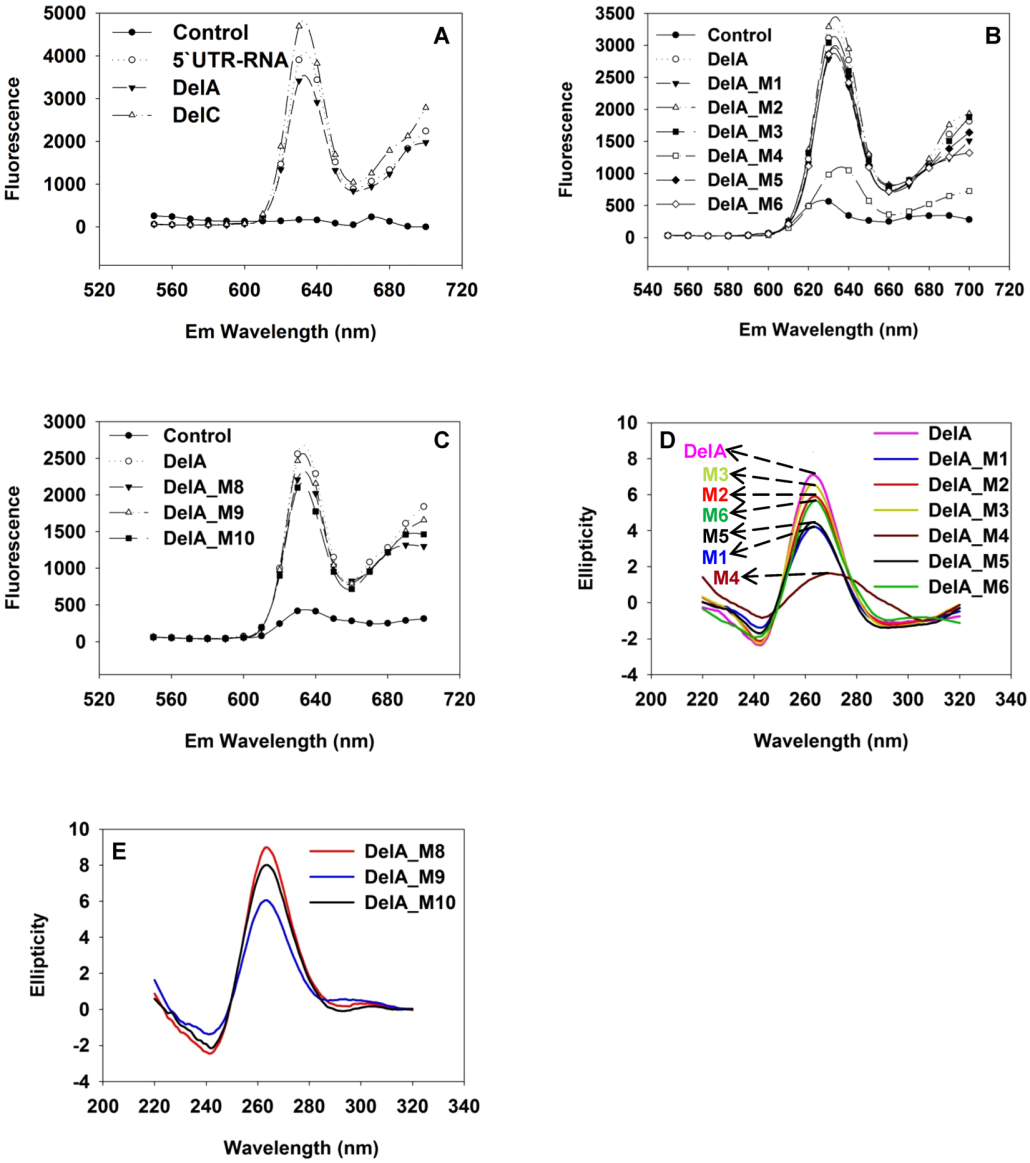
Characterization of the G-quadruplex within the constructed DNA oligos and P1-HNF4A-5′UTR RNA by PPIX-binding assay and CD spectra**. (A)** PPIX-binding assay of DelA, DelC, and the biosynthesized 89-nt P1-HNF4A-5′UTR RNA. **(B**,**C)** PPIX-binding assay of DNA oligos of all the mutant constructs of DelA. Controls in the PPIX-binding assay: 1 µM PPIX dissolved in 1X TE supplemented with 100 mM K^+^. **(D**,**E)** CD spectra of DNA oligos of DelA and all its mutants. The buffer for CD spectra is 5 mM Tris-HCl supplemented with 100 mM K^+^.

In order to verify that the G4 is formed in the P1-HNF4A-5′ UTR, we performed PPIX-binding assay using biosynthesized RNA oligo of the 5′UTR. PPIX is a G4-specific fluorescent probe that specially recognizes parallel G4s^41^. Binding of PPIX to the top/bottom of the G4 remarkably increases the solubility and fluorescence signal of PPIX. As expected, P1-HNF4A-5′ UTR RNA had a strong positive peak at 640 nm in PPIX-binding-assay (Fig. 3A), which is the typical signature of G4s^41^. This is a solid evidence of the *in vitro* presence of the G4 within the P1-HNF4A-5′UTR mRNA. Additionally, DelA and all its mutants (by using DNA oligos), except DelA_M4, had the similar G4 patterns (Fig. 3B & C). We also conducted CD spectrum to further confirm the results from PPIX binding assays. Likewise, all individual mutations of the G4 within DelA, except DelA_M4, showed a classical parallel G4 pattern with slightly varied peak heights in CD spectrum (Fig. 3D). Moreover, the binding-site mutants DelA_M8, DelA_M9, and DelA_M10 displayed parallel G4 signatures similar to DelA (Fig. 3E). Thus, the 5′UTR and most of the deletion/mutation oligos are capable of forming G4 structure *in vitro*. Importantly, DelA_M4 is the only oligo that largely loses the G4 pattern, indicating that the 5^th^ GGG (Nt 29-31) is most critical for G4 conformation. We also measured the melting temperature (Tm) of several oligos to evaluate the changes of the G4 stability caused by these deletion/mutations. In the presence of 5 mM K^+^, only DelA_M4/M5/M6, mutations in the 1^st^ and 5^th^ GGG, had prominent decreases of Tms (~10 °C). The rest of mutations in the G4 backbone and side chains did not alter the G4 chemical stability. These results further indicate the importance of the 1^st^ and the 5^th^ GGG for G4 conformation in DelA.

### Two regulatory single-nucleotide polymorphisms (rSNPs) within P1-HNF4A-5′ UTR have decreased inhibitory effects on reporter activities

**Figure 4:**
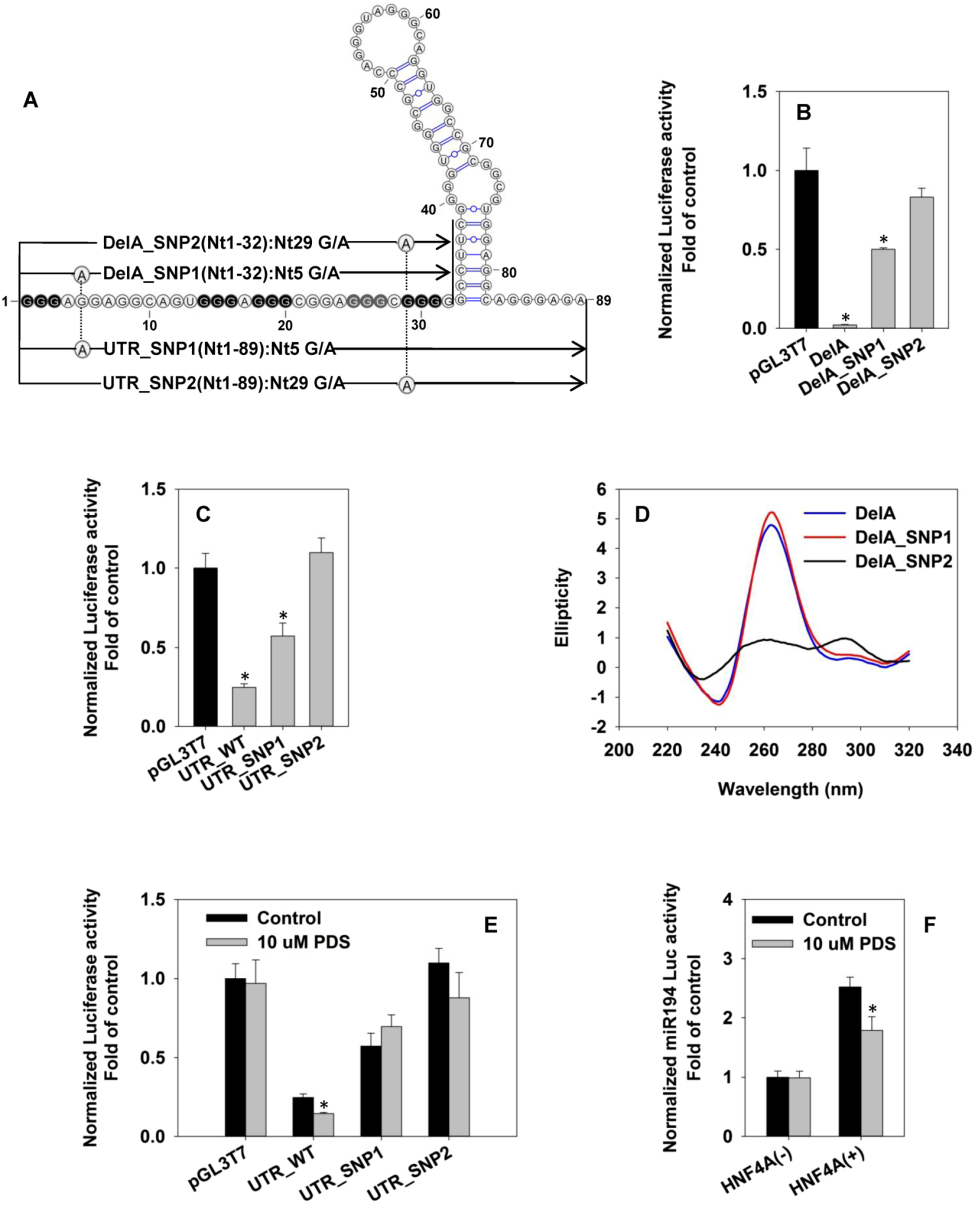
Characterization of two regulatory SNPs in the P1-HNF4A-5′ UTR. **(A)** Sequences and schematic structures of P1-HNF4A-5′UTR (UTR) and DelA that contains SNP1 and SNP2. **(B**,**C**) Dual-luciferase assay of reporters for SNP1 and SNP2 of P1-HNF4A-5′UTR (UTR) and DelA in HepG2 cells. *p < 0.05 versus pGL3T7 control. **(D)** CD spectra of DNA oligos of DelA, DelA_SNP1 and DelA_SNP2 **(E)** Effects of pyridostatin (PDS) treatment on the activities of luciferase reporters for wildtype (WT), SNP1, and SNP2 of P1-HNF4A-5′UTR (UTR_WT) in HEK293 cells. **(F)** Effect of PDS treatment on the activation of miR-194 reporter by pcDNA3-HNF4A1–5′UTR in HEK293 cells. N = 4, mean ± SD. *p < 0.05 versus vehicle control.

By examining the NCBI dbSNP database^42^, we found two rSNPs in P1-HNF4A-5′ UTR, namely rs546643401 (SNP1) and rs75356504 (SNP2) with single mutation at the predicted RBP-binding site and the G4 backbone, respectively. DelA_SNP1/SNP2 and UTR_SNP1/SNP2 were generated to elucidate the effects of the 2 SNPs on DelA and the full 5′ UTR (Fig. 4A). DelA_SNP1 (50% < control) and UTR_SNP1 (42% < control) had only moderate inhibitory effects, whereas DelA_SNP2 and UTR_SNP2 completely lost the inhibitory effects (Fig. 4B & C). In CD spectra, the RBP-binding-site-mutant DelA_SNP1 maintained the G4 pattern, whereas the mutation of the 5^th^ GGG in DelA_SNP2 largely abolished the G4 signature (Fig. 4D).

Pyridostatin (PDS) is a G4-specific ligand^43^. At 10 μM, PDS specifically decreased P1-HNF4A-5′ UTR reporter activity by 45% (Fig. 4E), and decreased the ability of HNF4A1-5′UTR to activate the miR-194 reporter by 30% (Fig. 4F). However, PDS treatment had no effect on the reporter activities of UTR_SNP1 and UTR_SNP2, indicating the resistance of the two SNPs to G4-stabilizing chemicals (Fig. 4E).

### G4 motifs within HNF3β, CCAAT/enhancer binding protein β (C/EBPβ), and nuclear receptor corepressor 1 (NCOR1) 5′UTRs have strong inhibitory effects on luciferase reporter activities in HepG2 cells

**Figure 5:**
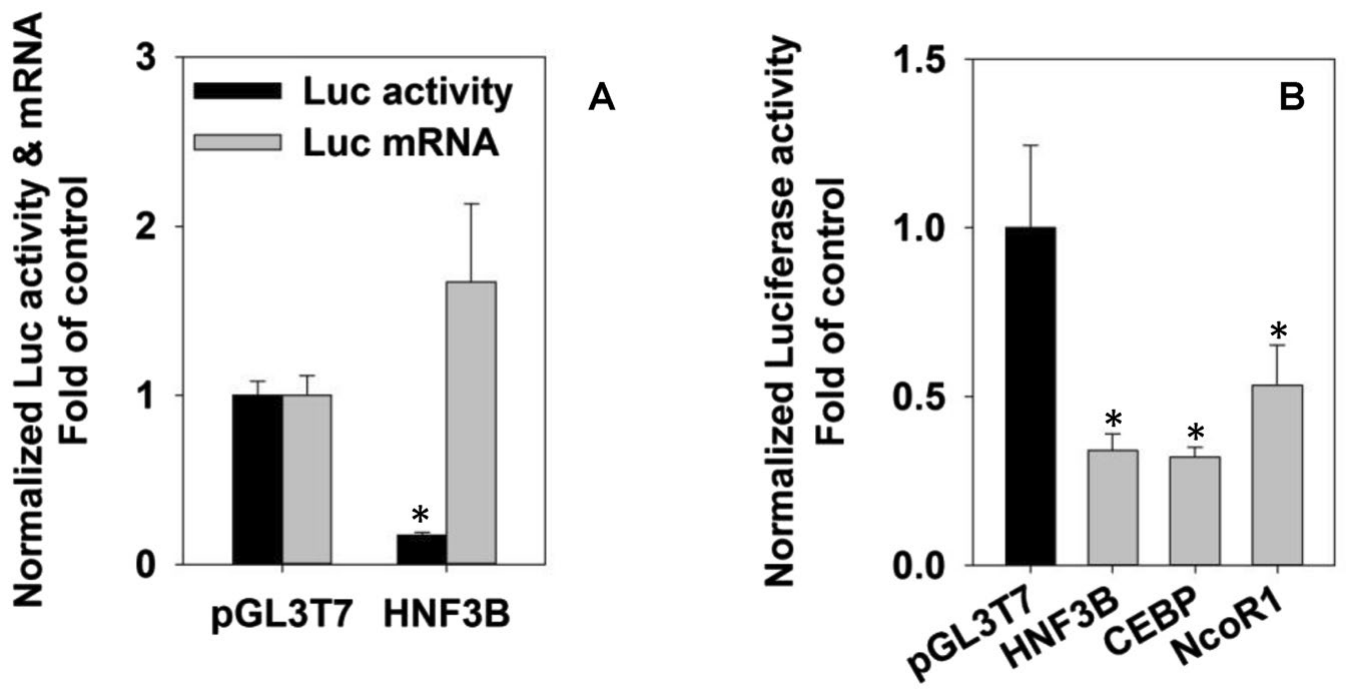
Characterization of G-quadruplex motifs in the 5′ UTRs of transcription factors. **(A)** Regulation of luciferase reporter activities by G-quadruplex motifs from the 5′ UTRs of transcription factors in HepG2 cells. **(B)** Luciferase activities and mRNA levels of luciferase reporters for the G-quadruplex motif in the 5′ UTR of HNF3B. N = 3, mean ± SD. *p < 0.05 versus the corresponding pGL3T7 control.

In addition to P1-HNF4A, we found strong inhibitory effects on the reporter activities by G4 motifs from 5′UTRs of several liver-enriched transcription factors (LETFs) including C/EBPβ (66% < control), NCOR1 (47% < control), and HNF3β (68% < control) (Fig. 5A). Similar to the G4s within the P1-HNF4A-5′ UTR, the G4 motif within the HNF3B-5′ UTR had no effect on the mRNA expression of the reporter gene (Fig. 5B). All inserted sequence is listed in Supplemental Table 1.

## Discussion

In the current study, we used multiple approaches to validate the *in vitro* presence of G4 in the 5′ UTR of human P1-HNF4A, a well-established tumor suppressor and master regulator of liver development and function. Data from multiple experiments strongly support a novel working model that the tight conjunction of RBP-binding sites and the adjacent G4 within P1-HNF4A-5′UTR is both necessary and sufficient to exert a strong inhibitory effect on the protein expression of P1-HNF4α. PDS, a G4-stabilizing ligand, can further specifically potentiate the translational suppressing effect of P1-HNF4A-5′UTR. Furthermore, we identified two rSNPs within P1-HNF4A-5′UTR that have partial/complete loss of the suppressing effect and are resistant to PDS.

**Figure 6:**
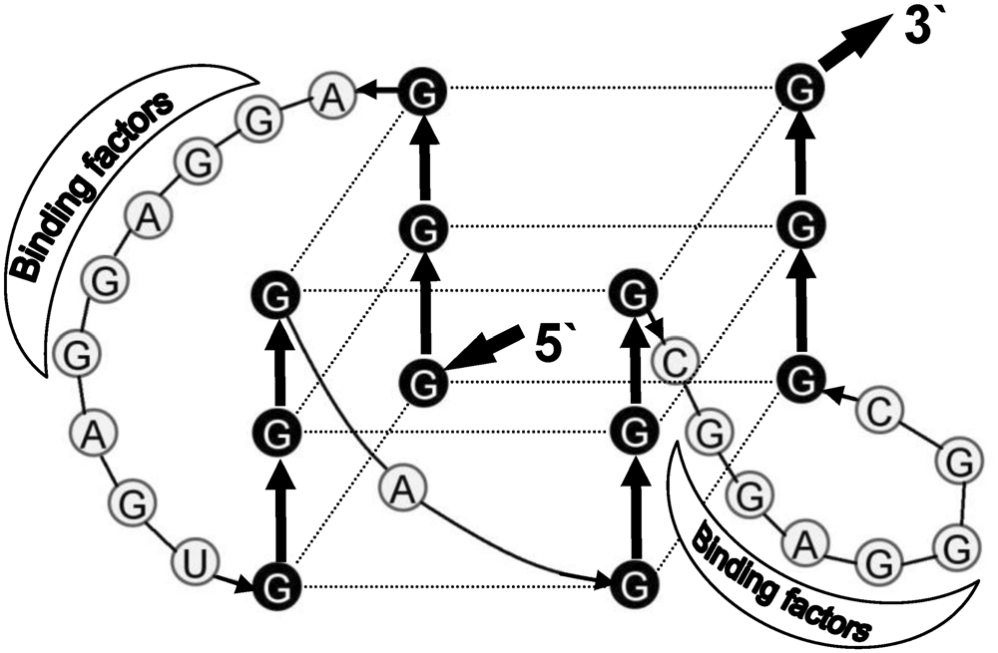
Hypothetical model of G-quadruplex (G4) formed in the P1-HNF4A-5′UTR. The bolded black arrows indicate the orientation of the 5′UTR. The guanines (Gs) in black constitute the G4 backbone. The two long side chains with the GGAGG core motif can recruit RNA-binding proteins (RBPs). The major binding factors predicted by RBPmap are as follows: HNRNPs (A1, A2B1, F, H1 and H2), RBM4 & 5, and SRSF1, 2 & 9. In the absence of those RBPs, the entire G4 is prone to be resolved by G4-unwinding factors. Conversely, binding of certain RBPs stabilizes the G4 within the 5′UTR, and consequently causes a strong translational repression. The G4-specific ligand PDS can bind to and stabilize G4 within the 5′UTR, resulting in enhanced translational repression by P1-HNF4A-5′UTR.

Overall, the SAR study of P1-HNF4A-5′ UTR proves that the major motif that causes the strong translational repression is Nt1-32, where both G4 and the potential RBP-binding sites are required to maintain the inhibitory effect (Fig. 2). Our SAR data suggest that the composition of the G4 within the 5′ UTR is as follows: **1)** the 5^th^ GGG (Nt29-31) is most critical in maintaining the G4 with strong inhibitory effect. Disruption of this GGG (DelA_M4 and DelA_SNP2) causes a complete loss of the inhibitory effect (Figs 2D & 4B) and a largely diminished G4 signature (Figs 3B, D, 4D); 2) the 1^st^ (Nt1-3), 2^nd^ (Nt14-16), and 3^rd^ (Nt18-20) GGG weigh comparably as their corresponding mutations completely abolishes the suppressing effect (Fig. 2D). However, these 3 GGG sets appear less critical than the 5^th^ GGG in the formation of G4 *in vitro* since the G4 signature is still maintained, suggesting that an alternative G4 can still be formed with the remaining GGG sets (Fig. 3B & D); 3) The 4^th^ GGG (Nt25-27) is the least important and likely, it is not used to form the major G4 backbone because the corresponding mutant (DelA_M1) still maintains a weak inhibitory effect (Fig. 2D). The largely diminished inhibitory effect by DelA_M1 might be the consequence of the alteration in the RBP-binding sites. Therefore, we propose that the major conformation of G4 within P1-HNF4A-5′UTR (Fig. 6) is using the 1^st^, 2^nd^, 3^rd^ and 5^th^ GGG based upon all the above indications, as well as the following additional data and information: **1)** The transcriptional event might favor the formation of G4 utilizing the 1^st^ GGGs once they are transcribed; **2)** DelA_M5 and DelA_M6, mutations in the 1^st^ GGG have reduced Tm (55 °C & 53 °C) compared to DelA (65 °C), indicating that the alternative G4 formed without the 1^st^ GGG is less stable; and **3)** DelA_M8 and DelA_M9 completely lose the inhibitory effect in HepG2 cells (Fig. 2F). It is thus unlikely that the major G4 is formed by 2^nd^ -5^th^ GGG, as in such case DelA_M8 and DelA_M9 would not compromise either the backbone or the side chains of the G4. Thus, a more reasonable explanation is that the predominant G4 is formed using the 1^st^ GGG, so the potential RBP-binding sites (M8 and M9) in the G4 side chains can play critical roles in stabilizing the entire G4 structure when bound with RBPs (Fig. 6).

To date, the regulation of the G4 by RBPs is largely unknown, but may involve G4 stabilizers and destabilizers^44^. The formation of G4 is dynamic^45^, and may be altered by the competition of distinct RBPs when bound to G4-forming motifs. Taken together, we establish a cooperative model for P1-HNF4A-5′UTR (Fig. 6): the major G4 formed within Nt1-32 has two long side-chains, which may recruit RBPs. This G4 conformation allows the G4 to survive *in vivo* from competitive binding of G4-unwinding factors, and exert the translational suppressing effects. It is not a surprise that G4 can cooperate with RBPs to modulate the mRNA translation. One example is that the direct interaction of HNRNPA1 with the G4 within the 5′UTR of RON/MTS1R (a tyrosine kinase receptor) mRNA activates the mRNA translation^46^. However, unlike the above co-regulatory model where the G4 and RBP-binding site are geographically separated^46^, the P1-HNF4A-5′ UTR appears to have a tight conjunction of G4 with the RBP-binding sites. In addition, G4 is known to be stabilized via consolidating the side chains. For instance, the quadruplex-duplex hybrids is a well known stable conformation that contains the stem loop as the side chain of G4, which shows a high stability^47^. Noteworthy, a recent study published in *Science* proposes that the RNA G4s are globally unfolded within eukaryotic cells, potentially with the assistance of RBPs, which are largely comparable with our predicted side-chain-binders: hnRNP families (A1, A2, F/H, D0), SRSF1/2 and CBF-A^48^. They speculate that in the G4-containing-5′ UTR-mediated translational repression, RBPs may unfold the G4 structure and remain bound to the 5′ UTR, which subsequently represses translation initiation. Our established model for P1-HNF4A-5′ UTR agrees with this proposed scenario in that the G4 is prone to be unfolded *in vivo* and the predicted RBPs remain bound to the 5′ UTR. However, we speculate that the final outcome of the G4 formation may be decided by the dominant binders (G4 stabilizer or destabilzer) and the binding loci (side chains or backbone). The present study does not provide definitive evidence of G4 formation *in vivo*. To date, the detection of G4 *in vivo* is very limited. The reverse transcriptase stalling caused by G4s has been used to detect G4 formation^48^. However, a previous primer extension assay, which was conducted to determine the length of the 5′UTR, indicates that the P1-HNF4A-5′UTR is unable to terminate the reverse transcription^34^. Although it was reported that the stalling of the reverse-transcription by many G4s are K^+^ dependent^48^, our additional data demonstrated that the P1-HNF4A-5′UTR did not cease the reverse transcription even under the physiological concentration of K^+^ (150 mM) (Fig. S2). Thus, this method may not be feasible to determine the formation of G4 in P1-HNF4A-5′UTR *in vivo*. Nevertheless, our data that the G4 ligand PDS specifically potentiates the translational inhibitory effects of P1-HNF4A-5′ UTR (Fig. 4E & F) provide a direct support that the RNA G4 is most likely formed within P1-HNF4A-5′ UTR in cells; in this case, binding of PDS to the G4 shifts the *in vivo* dynamic equilibrium of G4 folding-unfolding toward folding, and those RBPs bound to the G4 within the 5′ UTR mainly stabilize the entire G4 structure (Fig. 6).

The present study discovers two rSNPs, rs546643401 and rs75356504, within P1-HNF4A 5′UTR that may act as protective SNPs to reduce the individual susceptibility to liver cancer and other liver diseases via up-regulating P1-HNF4α. In this regard, a single site-directed mutation in the 5′ UTR of endogenous HNF4A gene may dramatically enhance the expression level of the P1-HNF4α protein. This may be explored as a novel therapy for liver cancer via gene editing. The recently developed CRISPR-Cas9 (Clustered regularly interspaced short palindromic repeats, CRISPR-associated protein 9) system is an efficient and simplified tool for genome engineering^49–52^. A recent successful application of CRISPR-Cas9 to correct genetic disorders in mouse hepatocyte has been reported^53^. Future study on the use of the CRISPR-Cas9 system to specifically enhance the protein expression of P1- HNF4α to treat liver cancer is warranted.

In addition to HNF4α, the tumor-suppressor p53 and a subset of LETFs also contain potential G4 motifs in the 5′ UTR (Supplemental Table 2). The present study confirms the translational inhibitory effects of G4 motifs within the 5′UTRs of HNF3β, NCOR1, and C/EBPβ (Fig. 5A) which all play critical roles in liver development and liver function. HNF3β is essential in liver development^54^, and it functions as a tumor-suppressor in liver^55^. C/EBPβ is down-regulated in human and mouse HCC, whereas its over-expression causes cell-cycle arrest in hepatoma cells^56,57^. NCOR1 is also reported to be down-regulated in HCC^58^. Thus, all these LETFs function as tumor-suppressors in human liver cancer. The potential roles of G4 motifs and RBPs in regulating the protein expression of these tumor-suppressors warrant further investigation.

Currently, how to improve the specificity of G4-interacting chemicals is a bottleneck in the development of novel anticancer drugs by stabilizing the G4 located within the promoter and 5′UTRs of oncogenes and the telomere^59^. Some of these G4-stabilizing small molecules, particularly the porphyrin analogs, have relatively high toxicity toward normal cells^60^. The present study, for the first time, reports the significance of G4 motifs in the 5′UTR of a key tumor suppressor, which uncovers a novel mechanism that G4-stabilizing compounds may cause cytotoxicity to normal cells: those compounds may also target on the G4 motifs within the 5′ UTRs to inhibit the protein expression of certain master regulators of cellular physiology (such as P1-HNF4α and other LETFs). The discovery of the presence of G4 in the 5′ UTR of P1-HNF4A and the inhibition of P1-HNF4α expression by the G4-specific ligand PDS may well promote the in-depth SAR studies of G4 and G4-interacting RBPs, particularly RBP–G4 RNA interaction interfaces^61^ which may help develop paradigm-shift approaches for cancer therapy by inhibiting oncogenes and/or increasing the expression of tumor-suppressors via specific modulation of the G4 and G4-interacting RBPs.

In conclusion, the present study provides the first evidence of the *in vitro* presence of G4 in the 5′ UTR of human P1-HNF4A. Multiple lines of evidence support our novel working model that the formation of a tight conjunction of G4 and the neighboring cis-elements in the 5′ UTR plays the key role in mediating the strong inhibition of protein expression by P1-HNF4A-5′ UTR. Potential inhibition of protein expression of P1-HNF4α due to stabilization of G4 in the 5′ UTR should be evaluated in the development of G4 ligands as anticancer drugs. Future in-depth SAR studies on the regulatory mechanisms of G4s by RBPs and the exploration of gene-editing technologies may greatly advance the basic research of gene regulation and the development of novel cancer therapies to target the “undruggable” oncogenes and tumor suppressors.

## Methods

### Plasmids construction

The sense strand (SS, 5′-**AGCT**GGGAGGAGGCAGT**GGG**A**GGG**CGGA**GGG**CG**GGG**GCCTTC**GGG**GT**GGG**CGCCCA**GGG**TA**GGG**CAGGTGGCCGCGGCGTGGAGGCA**GGG**AGA**C**-3′) and the anti-sense strand (AS, 5′-**CATGG**TCTCCCTGCCTCCACGCCGCGGCCACCTG CCCTACCCTGGGCGCCCACCCCGAAGGCCCCCGCCCTCCGCCCTCCCACTGCCTCCTCCC-3′) of P1-HNF4A wild-type (WT) 89-nt 5′ UTR^34^ were synthesized by Integrated DNATechnologies (IDT). Additional nucleotides marked in bold at the 5′ and 3′ terminals produce artificial HindIII and NcoI sites. The annealed 89-nt wild-type and mutant P1-HNF4A-5′ UTR were cloned into the HindIII/NcoI sites of pGL3T7 vector, a modified backbone based on pGL3-promoter (Promega) that contains a T7 promoter to drive the *in-vitro*-transcription/translation. The inserted 5′ UTRs were immediately upstream of the translation start site of the luciferase cDNA. The newly constructed vectors were named as pGL3T7-HNF4A-5′UTR. All other reporter vectors for SNPs and deleted/mutated fragments were constructed by the same method. The sequence information of all deletion/mutation constructs is provided in Supplemental Table 1. To create the HNF4A1 expression vector with 5′ UTR, we inserted the annealed 5′ UTR into the backbone of pcDNA3-HNF4A1-cDNA (a gift from Dr. Todd Leff)^62^. The newly created construct was named as pcDNA3-HNF4A1-5′UTR. All the constructed expression and reporter vectors were verified by sequencing.

### Transient transfection and dual-luciferase assay

HEK293 and HepG2 cells were cultured with MEM medium (Corning) supplemented with 10% fetal calf serum. Twenty-four hours after seeding, transfection was conducted using Lipofectamine 3000 (Invitrogen), following the manufacturer’s protocol. In the 96-well-plate, each well were transfected with firefly luciferase vectors, the control renilla luciferase vector pRL-CMV, and/or the HNF4A1 expression vector. Twenty-four hours after transfection, cells were harvested for dual-luciferase assay using Dual-Glo™ luciferase assay system (Promega) and GloMax Luminometer (Promega), following the manufacturer’s protocol. The ratios of firefly/renilla luciferase activities were calculated as the normalized reporter activity, with the control values set at 1.0. For treatment of the G4-specific ligand, pyridostatin (PDS, Sigma) in aqueous solution was added 18 h after transfection of HEK293 cells^63^, and HEK293 cells were harvested for dual-luciferase assay 6 h after PDS treatment.

### Western blot

HEK293 cells in 6-well-plates were transfected with 500 ng HNF4α expression vectors and 150 ng pcDNA3-eGFP. Whole cell lysates were prepared 24 h after transfection. Proteins in cell lysates were resolved in sodium dodecyl sulphate-polyacrylamide gel electrophoresis. Western blot quantification of HNF4α and EGFP was conducted with primary antibodies as follows: anti-HNF4α (H1415, PPMX) and anti-GFP (ab290, Abcam). Primary antibodies were revealed with HRP-conjugated secondary antibodies (Anti-mouse IgG, #7076; Anti-rabbit IgG, #7074, Cell Signaling) and ECL Western Blotting Substrate (W1015, Promega). ChemiDoc^TM^ XRS + System (Bio-Rad) and Image-J software were used for band capturing and density analysis.

### Real-time PCR

Total RNAs from transfected cells in 6-well-plate were isolated by RNA-STAT60 (Tel-Test) and quantified by Qubit RNA assay kit and Qubit 2.0 fluorometer (Life technology). One µg of RNA was reverse transcribed using the High-Capacity RNA-to-cDNA^TM^ Kit (Applied Biosystems®, life technologies) for cDNA synthesis, following the manufacturer’s instructions. iQ™ SYBR® Green Supermix (Bio-Rad) was applied to quantify mRNAs using MyiQ2™ Two-Color Real-Time PCR Detection System (Bio-Rad). The amounts of mRNA were calculated using the comparative CT method, which determines the amount of target gene normalized to an introduced control (e.g. EGFP or renilla luciferase). The sequences of real-time PCR primers (synthesized by IDT) were listed in Supplemental Table 3.

### PPIX-binding assay

DNA/RNA fragments (2 µM in TE buffer) were heated at 88 °C for 4 min and then cooled down to room temperature. An equal volume of PPIX solution (2 µM) coupled with 200 mM KCl in TE buffer was then mixed with DNA/RNA oligos (the final concentration of DNA/RNA-PPIX complex is 1 µM in 100 mM K^+^). The resultant mixtures were incubated in the dark for 2 h at room temperature, followed by the fluorescence scanning using synergy micro-plate reader (BioTek). The Ex wavelength was fixed at 410 nm and the Em wavelength varied from 550 to 700 nm.

### CD Spectrum

All DNA oligos (4 µM) were dissolved in Buffer A (5 mM Tris-HCl, pH 7.5, 100 mM KCl) for wavelength scanning and Buffer B (5 mM Tris-HCl, pH 7.5, 5 mM KCl) for melting temperature (Tm) determination. The DNA solution was heated at 90 °C × 10 min and gradually cooled down to 25 °C in 40 min. All data were collected by Aviv Model 410 CD spectrometer (Aviv Biomedical). The CD spectra of oligos were scanned from 220 to 320 nm at 25 °C. To determine the Tm of G4s, temperature-dependent (25 °C-88 °C) changes in the CD of oligos were monitored at 260 nm. Each measurement of DNA oligo uses its corresponding buffer as the background, which was subtracted in the final data analysis.

### *In vitro* transcription and translation

Two µg plasmid of pGL3T7-HNF4A-5′UTR was linearized by NcoI digestion and isolated by Gene Jet Extraction and DNA Clean-up Kit (Fisher) for *in vitro* transcription using MEGA script T7 kit (Ambion). The synthesized transcript was isolated by RNA-STAT60 (Tel-Test) and quantified by Qubit RNA assay kit and Qubit 2.0 fluorometer (Life technology). TNT® Quick Coupled Transcription/Translation System (Promega) was used for *in vitro* translation. Briefly, template plasmids (125 ng firefly luciferase vectors coupled with 125 ng pRL-CMV) were mixed with TNT® T7 Quick Master Mix and methionine (25 µM). The whole mixture was incubated at 30 °C for 90 min, after which 1 µl reaction products were used for dual-luciferase assay.

### Statistical analysis

All values were expressed as mean ± S.D. For comparison of two groups, the two-tailed student’s t-test was used to determine the statistical difference, which was set at p < 0.05. For multiple comparisons, analysis of variance (ANOVA) was performed, followed by the Student-Newman-Keuls Method in SigmaPlot 12.5, with significance set at p < 0.05.

### Data availability

All supplementary data is accessible on nature.com.

## Electronic supplementary material

Supplemental information
