# Supplementary material for: Conjunction of potential G-quadruplex and adjacent cis-elements in the 5′ UTR of hepatocyte nuclear factor 4-alpha strongly inhibit protein expression
Source: Sci Rep. 2017 Dec 12;7:17444. doi: 10.1038/s41598-017-17629-y (PMC5727235; doi:10.1038/s41598-017-17629-y)
Supplement: Supplementary file 1 — Supplemental information [file 41598_2017_17629_MOESM1_ESM.pdf]

Conjunction of potential G-quadruplex and adjacent cis-elements in the 5' UTR of hepatocyte nuclear factor 4-alpha strongly inhibit protein expression

Shangdong Guo[1] and Hong Lu[1,*]

[1] Department of Pharmacology, SUNY Upstate Medical University, Syracuse, NY 13210, U.S.

* To whom correspondence should be addressed. Tel: 315-464-7978; Fax: 315-464-8008;
Email: luh@upstate.edu.

Table. 1. Sequences of deletion/mutation reporter constructs for P1-HNF4A-5' UTR.

Constructs	Sequence
**UTR_WT**	GGGAGGAGGCAGTGGGAGGGCGGAGGGCGGGGGCCTTCGGGGTGGGCGCCCAGGGTAGGGCAG GTGGCCGCGGCGTGGAGGCAGGGAGA
**UTR_9G_Mut**	GGGAGGAGGCAGTGTGAGTGCGGAGTGCGGTGGCCTTCGTGGTGTGCGCCCAGTGTAGTGCAGGT GGCCGCGGCGTGGAGGCAGTGAGA
**UTR_SNP1**	GGGAGGAAGCAGTGGGAGGGCGGAGGGCGGGGGCCTTCGGGGTGGGCGCCCAGGGTAGGGCAG GTGGCCGCGGCGTGGAGGCAGGGAGA
**UTR_SNP2**	GGGAGGAGGCAGTGGGAGGGCGGAGGGCAGGGGCCTTCGGGGTGGGCGCCCAGGGTAGGGCAG GTGGCCGCGGCGTGGAGGCAGGGAGA
**DelA**	GGGAGGAGGCAGTGGGAGGGCGGAGGGCGGGG
**DelA_M1**	GGGAGGAGGCAGTGGGAGGGCGGAGAGCGGGG
**DelA_M2**	GGGAGGAGGCAGTGAGAGGGCGGAGGGCGGGG
**DelA_M3**	GGGAGGAGGCAGTGGGAGAGCGGAGGGCGGGG
**DelA_M4**	GGGAGGAGGCAGTGGGAGGGCGGAGGGCGAAG
**DelA_M5**	GGTAGGAGGCAGTGGGAGGGCGGAGGGCGGGG
**DelA_M6**	GGCAGGAGGCAGTGGGAGGGCGGAGGGCGGGG
**DelA_M7**	GGGAGGAGGCAGTGGGAGAGCGGAGAGCGGGG
**DelA_M8**	GGGTGGAGGCAGTGGGAGGGCGGAGGGCGGGG
**DelA_M9**	GGGAGGTGGCAGTGGGAGGGCGGAGGGCGGGG
**DelA_M10**	GGGAGGAGGCAGTGGGAGGGCGGTGGGCGGGG
**DelA_SNP1**	GGGAGGAAGCAGTGGGAGGGCGGAGGGCGGGG
**DelA_SNP2**	GGGAGGAGGCAGTGGGAGGGCGGAGGGCAGGG
**DelB**	GCCTTCGGGGTGGGCGCCCAGGGTAGGGCAGGTGGCCGCGGCGTGGAGGCAGGGAGA
**DelC**	GGGAGGGCGGAGGGCGGGG
**C/EBPβ**	GGGCGGGGGTGGGCAGGG
**HNF3β**	GGGTGGGGGTGGGGGG
**NCOR1**	GGGCTGGGGGGAGGGAGAGGG

Note. The sites of mutations are in the black shade.

Table. 2. List of genes with putative 3-ring or 4-ring G-quadruplex (G4) motifs in their 5' UTR.

Gene	Accession No./ Transcript ID	5`UTR length	G4 position	G4 Length	Quadruplex forming G-Rich Sequence(QGRS)	G Score
**HNF1α**	**NM_001306179.1**	**202**	**2**	**32**	GGGCCCTGATTCACGGGCCGCTGGGGC CAGGG	**65**
**HNF1α**	**NM_001306179.1**	**202**	**36**	**45**	GGGGGTTGGGGGTGCCCACAGGGCTTG GCTAGTGGGGTTTTGGGG	**91**
**HNF3β**	**NM_021784.4**	**185**	**47**	**37**	GGGAGTGGAGCCCAGGGAGAGGGAGC GCGAGAGAGGG	**64**
**HNF3β**	**NM_021784.4**	**185**	**127**	**16**	GGGTGGGGGTGGGGGG	**70**
**C/EBP α**	**NM_004364.4**	**125**	**52**	**26**	GGGCGCGGGCGAGCAGGGTCTCCGGG	**69**
**C/EBP β**	**NM_005194.3**	**451**	**20**	**18**	GGGCGGGGGTGGGCAGGG	**72**
**C/EBP β**	**NM_005194.3**	**451**	**268**	**28**	GGGACTGGGAAGGGGACCCACCCGAGG G	**65**
**HDAC3**	**NM_003883.3**	**66**	**6**	**43**	GGGCTGGCGGCGGCCGCGGGCGGCGG GCGGCGGAGGTGCGGGG	**62**
**NCOR1**	**NM_006311.3**	**269**	**3**	**21**	GGGCTGGGGGGAGGGAGAGGG	**72**
**P53**	**NM_000546.5**	**203**	**86**	**44**	GGGAGCAGGTAGCTGCTGGGCTCCGG GGACACTTTGCGTTCGGG	**63**

Table. 3. List of primers for real-time-PCR

Primer name	Sequence	Accession number /Vector name	Amplicon length
Fire_Luc_for	CTGGAGAGCAACTGCATAAGG	pGL3-promoter	142 bp
Fire_Luc_rev	CGTTTCATAGCTTCTGCCAAC	pGL3-promoter	
Renilla_Luc_for	TGTGCCACATATTGAGCCAGTA	pRL-CMV	134 bp
Renilla_Luc_rev	GAAGTTCAAACCATGCAGTAAGA	pRL-CMV	
eGFP_for	ACATGAAGCAGCACGACTTCT	pcDNA3-EGFP	227 bp
eGFP_rev	GATATAGACGTTGTGGCTGTTG	pcDNA3-EGFP	
HNF4A_for	GATCAGCACTCGAAGGTCAAG	NM_178849.2	171 bp
HNF4A_rev	TCCTTCATGGACTCACACACA	NM_178849.2	
HNF4A_UTR_for	GCTAACTAGAGAACCCACTGCTTACTGGC	pcDNA3-HNF4A1-5`UTR	350bp
HNF4A_UTR_rev	CATCGTCAACACCTGCACATT	pcDNA3-HNF4A1-5`UTR	

Figure. S1. Original image of the western blot

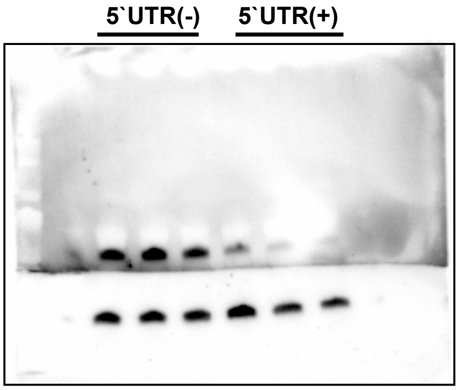

A

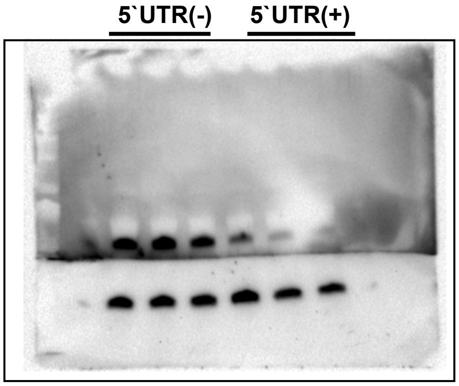

B

Figure S1. (A) & (B) Western blot of over-expressed HNF4α and eGFP with 2 different exposures. HNF4A expression vectors with/without 5`UTR and an EGFP expression vector were co-transfected into HEK293 cells. The displayed bands come from two sections of a single gel. N=3, mean ± SD.

Figure. S2. Effect of potassium concentrations on the reverse transcription of P1-HNF4A-5`UTR RNA

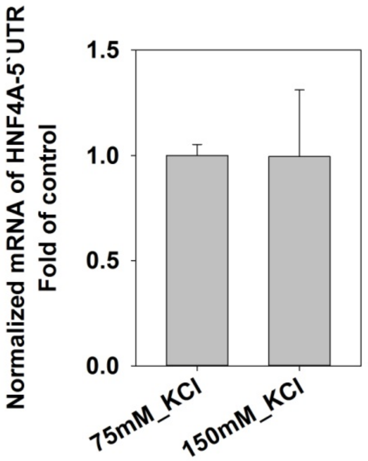

Figure S2. Effects of potassium concentrations on the reverse transcription of P1-HNF4A-5`UTR RNA. The reverse transcription of P1-HNF4A-5`UTR RNA was conducted with 75 mM and 150 mM KCl. Amounts of P1-HNF4A-5`UTR cDNA were quantified by real-time PCR and normalized to the co-transfected Renilla Luciferase. N=3, mean ± SD.

Method

Reverse Transcription and real-time-PCR determination of P1-HNF4A-5'UTR RNA

HEK293 cells were seeded in 6-well-plate. Twenty-four hours after seeding, pcDNA3-HNF4A1-5`UTR and pRL-CMV vectors were co-transfected into cells using lipofectamine 3000 (Invitrogen), per manufacturer's protocol. Twenty-four hours after transfection, total RNAs were isolated by RNA-STAT60 (Tel-test) and quantified by Qubit RNA assay kit and Qubit 2.0 fluorometer (Life technology). Total RNAs (250 ng) were treated with RNAse-free DNAse and then reverse transcribed using SuperScript™III reverse transcriptase (Invitrogen) with following conditions: 42°C, 75 mM KCl/150 mM KCl, 60 min. iQ™ SYBR® Green Supermix (Bio-Rad) was applied to quantify mRNAs using MyiQ2™ Two-Color Real-Time PCR Detection System (Bio-Rad). The amounts of mRNA were calculated using the comparative CT method, which determines the amounts of P1-HNF4A-5`UTR normalized to the renilla luciferase. The forward primer for P1-HNF4A-5`UTR targets the sequence immediately downstream of the transcriptional start site of pcDNA3, and the reverse primer for P1-HNF4A-5`UTR targets the 5' sequence of HNF4A1 cDNA. All real-time PCR primers (synthesized by IDT) were listed in Supplemental Table 3.

Figure. S3. Effect of potassium concentrations on the *in vitro* translation of pGL3T7-HNF4A-5`UTR.

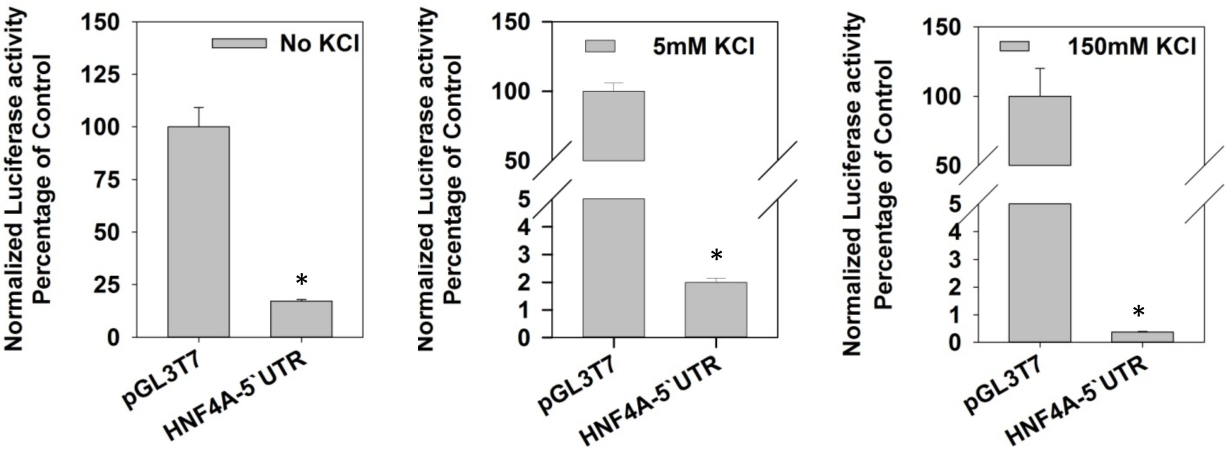

Figure S3. Effect of potassium concentrations on the *in vitro* translation of pGL3T7-HNF4A-5`UTR. N=3, mean ± SD. * $p < 0.05$ versus pGL3T7 group. In the presence of 0, 5, and 150 mM exogenous K^+, the P1-HNF4A-5'UTR caused 20%, 98.0%, and 99.6% inhibition of the luciferase reporter activities, respectively. These results suggest that the *in-vitro*-translation system has much weaker capability to unwind the G4 under a K^+ concentration that is identical with the cellular condition (150 mM).

Method

***In vitro* transcription and translation**

Two µg plasmid of pGL3T7-HNF4A-5`UTR was linearized by NcoI digestion and isolated by Gene Jet Extraction and DNA Clean-up Kit (Fisher) for *in vitro* transcription using MEGA script T7 kit (Ambion). The synthesized transcript was isolated by RNA-STAT60 (Tel-Test) and quantified by Qubit RNA assay kit and Qubit 2.0 fluorometer (Life technology). TNT® Quick Coupled Transcription/Translation System (Promega) was used for *in vitro* translation. Briefly, template plasmids (125 ng firefly luciferase vectors coupled with 125 ng pRL-CMV) were mixed with TNT® T7 Quick Master Mix and methionine (25 µM), with the addition of 0, 5, and 150 mM KCl (final concentration). The whole mixture was incubated at 30°C for 90 min, after which 1 µl reaction products were used for dual-luciferase assay.
